# Effects of Relative Magnetic Field, Chemical Reaction, Heat Generation and Newtonian Heating on Convection Flow of Casson Fluid over a Moving Vertical Plate Embedded in a Porous Medium

**DOI:** 10.1038/s41598-018-36243-0

**Published:** 2019-01-23

**Authors:** Dolat Khan, Arshad Khan, Ilyas Khan, Farhad Ali, Faizan ul Karim, I. Tlili

**Affiliations:** 1 Department of Mathematics, City University of Science and Information Technology, Peshawar, 25000 Pakistan; 2 Institute of Business and Management Sciences, The University of Agriculture, Peshawar, Khyber Pakhtunkhwa Pakistan; 3 Department of Mathematics, College of Science Al-Zulfi, Majmaah University, Al-Majmaah, 11952 Saudi Arabia; 4 Faculty of Mathematics and Statistics, Ton Duc Thang University, Ho Chi Minh City, Vietnam; 5 Computational Analysis Research Group, Ton Duc Thang University, Ho Chi Minh City, Vietnam; 6 Energy and Thermal Systems Laboratory, National Engineering School of Monastir, Street Ibn El Jazzar, 5019 Monastir, Tunisia

## Abstract

The aim of this article is to study the combined effects of heat generation and chemical reaction on magnetohydrodynamic (MHD) natural convection flow over a moving plate embedded in a porous medium. Natural convection is caused due to buoyancy forced which has been induced because of temperature and concentration gradients. The general condition of velocity has been considered on the plate surface with Newtonian heating and constant wall concentration. The effect of thermal radiation is also considered in the energy equation. The main objective here is to study the relative behavior of the magnetic field. That is the magnetic field shows two types of relative behavior. More exactly, when the magnetic field is fixed relative to the fluid (MFFRF) and the magnetic field is fixed relative to the plate (MFFRP). The general exact solution of the problem is determined by the Laplace transform method. Particular solutions for two special cases namely the plate with variable vibration and the plate with sine and cosine oscillations are also determined. Moreover, the solutions when *ζ* → ∞ for both cases i.e. MFFRF and MFFRP are also obtained as special cases. The velocity profile is presented in the form of mechanical, thermal and concentration components. Velocity obtained for oscillating plate condition is written in terms of steady-state and transient parts. Exact solutions obtained in this paper are interpreted graphically using computational software Mathcad-15 to examine the effects of various pertinent parameters such as Casson fluid parameter, the permeability of porous medium, chemical reaction parameter, heat generation parameter, buoyancy force parameter, magnetic parameter, and radiation parameter. Results for Sherwood number, skin-friction, and Nusselt number are numerically computed and discussed.

## Introduction

Previous work on the MHD natural convection flow with mass and heat transfer in electrically conducting fluid had taken much attention in the literature because of their wide applications in the field of electrical power generation, meteorology, solar physics, chemical engineering, and geophysics. Under different boundary conditions, there are some exact solutions for such motion of viscous incompressible fluids over an infinite vertical plate. Adrian Bejan^1^ showed a thermodynamic design for heat and mass transfer devices and processes. The effects of thermal and radiation diffusion on free convection MHD flow of a viscous incompressible fluid close to an oscillating plate set in a porous medium is analyzed by Khan *et al*.^2^. The aim of their work was to study the thermal and radiation diffusion effects on free convection MHD flow of viscous fluid. The knowledge of thermal transport of MHD non-Newtonian fluid flow over a melting sheet in the presence of exponential heat source was studied by Sandeep^3^. He obtained the numerical solution via fourth-order Runge–Kutta based shooting technique. Sandeep^4^ extended his previous work and analyzed the effects of radiation and rotation on unsteady hydromagnetic free convection flow of a viscous incompressible electrically conducting fluid past an impulsively moving vertical plate in a porous medium by applying inclined magnetic field via Laplace technique. The impact of frictional heating on MHD radiative ferrofluid is studied by Kempannagari *et al*.^5^. The MHD flow and heat transfer of two distinct non-Newtonian fluids across a stretching sheet are investigated by Ramandevi *et al*.^6^. Furthermore, Kumar *et al*.^7^ studied the problem of boundary layer flow of MHD electrically conducting fluid past a cone. The influence of cross diffusion on MHD flow of non-Newtonian fluids past a stretching sheet is studied by Reddy *et al*.^8^. Ananth *et al*.^9^ investigated the influence of nonlinear thermal radiation on stagnation point flow of a Casson fluid towards a stretching sheet. Recently, Lakshmi *et al*.^10^ investigated cross diffusion on MHD flow of Casson and Walters-B nanofluids past a variable thickness sheet. Shehzad *et al*.^11^ examined the effects of chemical reaction, mass transfer, and MHD flow on Casson fluid in a porous medium. Kataria *et al*.^12^ investigated analytically the influence of thermal radiation with chemical reaction on Casson fluid with MHD flow. The fluid was taken over an oscillating perpendicular plate with uniform and transverse magnetic fields. They obtained exact solutions via the Laplace transform method.

Casson fluid model was introduced by Casson in 1959, after that the flow characteristics of Casson fluids in tubes was first studied by Oka^13^. The examples of Casson fluid are of the type are as follows: jelly, tomato sauce, honey, soup, concentrated fruit juices, etc. Fung^14^ used human blood as a Casson fluid. Influence of magnetic field on peristaltic flow of a Casson fluid in an asymmetric channel was studied by Akbar^15^. The influence of a magnetic field on a Casson fluid past an oscillating vertical plate embedded in a porous medium was studied by Reddy *et al*.^16^. Three dimensional Casson fluid with MHD flow over a porous sheet has been studied by Nadeem *et al*.^17^. Mohyud-Din *et al*.^18^ studied the influence of nonlinear radiation on Casson fluid between parallel disks. Effect of the magnetic field on Casson fluid flow between parallel plates was studied by Ahmad *et al*.^19^. The influence of Newtonian heating on unsteady hydromantic Casson fluid flow was investigated by Das *et al*.^20^ with the effects of heat and mass transfers.

In many practical problems, the heat transfer from the plate surface is proportional to the local surface temperature, known as Newtonian heating (NH) effect^21^. Because of the interesting and practical applications of NH problems, scientists are attracted to take the NH condition in their studies. Merkin^22^ took NH condition and investigated the boundary layer natural convection flow over a vertical surface. Hussanan *et al*.^23^ obtained the exact solution of mass and heat transfer through a vertical plate with the effect of NH. In another article, Hussanan *et al*.^24^ explored the influence of heat transfer for unsteady boundary layer flow of Casson fluid with NH boundary condition. They solved these problems analytically by using the Laplace transform method. Recently, the influence of thermal radiation and heat transfer for unsteady Casson fluid with NH boundary condition is observed by Hussanan *et al*.^25^. They obtained the exact solution and discussed various parameters graphically. Furthermore, Vieru *et al*.^26^ studied analytically the effect of NH on the MHD flow of natural convection in the presence of mass diffusion. Recently, Fetecau *et al*.^27^ provided a general study of such kind of flow with the effect of radiative, heat source and stress on boundary. However, in all the above studies and previously published other works, the force of magnetic lines are fixed to the fluid. Lately, Narahari and Debnath^28^ investigated the MHD, free convection flow over a vertical plate with heat flux, absorption or generation when the force of the magnetic line is fixed to the fluid or plate. That is the magnetic field was taken in relative status. When magnetic field is fixed to the fluid or plate, play an important role in manufacturing industries for the design of fins, gas turbines nuclear, power plants, steel rolling and various propulsion devices for aircraft, satellites. missiles, furnace, and combustion design, energy utilization, materials processing, remote sensing for astronomy, temperature measurements and space exploration, cryogenic engineering and food processing, as well as numerous health, agricultural and military applications^29–31^. The solutions are obtained exactly and exponentially or constantly accelerating plate. Tokis^32^ was the first who obtained the exact solution of this type of problem. He discussed the solution by uniform, constantly acceleration or decaying of the plate. Onyango *et al*.^33^ investigated the force of the magnetic field is fixed to the fluid or plate for Couette flow among two parallel plates numerically.

From the above discussion, it is crystal clear that the effect of NH condition for Casson fluid moving over a vertical plate when the magnetic field show relative behavior with additional consequence of thermal radiation, porous medium, chemical reaction and heat generation is not investigated so far. Therefore, the objective of the current work is to make such an attempt. Exact solution of the problem is obtained for general velocity condition at the wall using the Laplace transform method with some interesting cases of particular solutions. More exactly, the influence of MHD is determined when MFFRF and MFFRP. Results are computed numerically via computational software MATHCAD-15 and plotted in various graphs.

## Mathematical Modeling and Solution

Let suppose an electrically conducting, unsteady free convection incompressible flow of Casson fluid over an infinite perpendicular plate. The magnetic field shows a relative behavior, i.e. MFFRF and MFFRP. The effects of chemical reaction and thermal radiation are also taken. Initially the plate and fluid are at rest with constant concentration and temperature *C*_∞_, *T*_∞_. At *t*^+^, the plate start to slide in its own plane beside the gravitational field *g* with the velocity *Uf*(*t*)with NH condition.

Maxwell equations state:

1 $${\rm{div}}{\bf{B}}={\bf{0}},\,{\rm{Curl}}{\bf{E}}={\boldsymbol{-}}\,\frac{\partial {\bf{B}}}{\partial t},\,{\rm{Curl}}{\bf{B}}={\mu }_{e}{\bf{J}}.$$

By using Ohm’s law

2 $${\bf{J}}=\sigma ({\bf{E}}+{\bf{V}}\times {\bf{B}}).$$

The magnetic field **B** is taken normal to **V**. The Reynolds number is so small that flow is laminar. Hence,

3 $$\frac{1}{\rho }{\bf{J}}\times {\bf{B}}=\frac{\sigma }{\rho }[({\bf{V}}\times {{\bf{B}}}_{0})\times {{\bf{B}}}_{0}]=-\,\frac{\sigma {B}_{0}^{2}{\bf{V}}}{\rho }.$$

The rheological equation for an incompressible flow of a Casson fluid can be written as, (see^24,25^)

4 $$\tau ={\tau }_{0}+\mu {\gamma }^{\cdot }$$

or

5 $${\tau }_{ab}=\{\begin{array}{ll}2({\mu }_{\eta }+\frac{{p}_{\lambda }}{\sqrt{2\pi }}){e}_{ab}, & \pi  > {\pi }_{c}\\ 2({\mu }_{\eta }+\frac{{p}_{\lambda }}{\sqrt{2{\pi }_{c}}}){e}_{ab}, & \pi  > {\pi }_{c}\end{array}.$$

Under these conditions and the concentration, temperature and velocity are the functions of (*y*, *t*)only, then the governing equations are reduced to the set of partial differential equations.

6 $$\begin{array}{rcl}\frac{\partial u(y,t)}{\partial t} & = & \nu (1+\frac{1}{\beta })\frac{{\partial }^{2}u(y,t)}{\partial {y}^{2}}-\frac{\sigma {B}_{0}^{2}}{\rho }u(y,t)-(1+\frac{1}{\beta })\frac{\nu \phi }{K}u(y,t)\\  &  & +\,g{\beta }_{{\rm{\Theta }}}({\rm{\Theta }}-{{\rm{\Theta }}}_{\infty })+g{\beta }_{c}(C-{C}_{\infty });y,t > 0.\end{array}$$

Equation (6) is valid if the magnetic field is fixed while for the relative magnetic field the Eq. (6) becomes (Narahari and Debnath^28^, Shah *et al*.^34^):

7 $$\begin{array}{rcl}\tfrac{\partial u(y,t)}{\partial t} & = & \nu (1+\tfrac{1}{\beta })\tfrac{{\partial }^{2}u(y,t)}{\partial {y}^{2}}-\tfrac{\sigma {B}_{0}^{2}}{\rho }(u(y,t)-\varepsilon Uf(t))-(1+\tfrac{1}{\beta })\tfrac{\nu \phi }{K}u(y,t)\\  &  & +\,g{\beta }_{{\rm{\Theta }}}({\rm{\Theta }}-{{\rm{\Theta }}}_{\infty })+g{\beta }_{c}(C-{C}_{\infty });y,t > 0,\end{array}$$

8 $$\rho {c}_{p}\frac{\partial {\rm{\Theta }}\,(y,t)}{\partial t}={k}_{1}\frac{{\partial }^{2}{\rm{\Theta }}\,(y,t)}{\partial {y}^{2}}-Q\,({\rm{\Theta }}-{{\rm{\Theta }}}_{\infty }),$$

9 $$\frac{\partial C(y,t)}{\partial t}=D\frac{{\partial }^{2}C(y,t)}{\partial {y}^{2}}-R(C-{C}_{\infty }),$$

with the initial and boundary conditions:

10 $$\begin{array}{l}u(y,t)=0,\,{\rm{\Theta }}(y,t)={{\rm{\Theta }}}_{\infty },\,C(y,t)={C}_{\infty };\,y\ge 0,\,t=0\\ u(y,t)=Uf(t),\,\frac{\partial {\rm{\Theta }}(y,t)}{\partial y}=-\,{h}_{s}{\rm{\Theta }},\,C(y,t)={C}_{w};\,t\ge 0,\,y=0\\ u(y,t)\to 0,\,{\rm{\Theta }}(y,t)\to {{\rm{\Theta }}}_{\infty },\,{\rm{C}}(y,t)\to {C}_{\infty }\,{\rm{as}}\,y\to \infty \end{array}\}.$$

Here *C*(*y*, *t*), *u*(*y*, *t*) Θ (*y*, *t*) and are the concentration, velocity, and temperature. *β*, *ν*, *σ*, *B*_0_, *ρ*, *ϕ*, *K*, *k*_1_, *g*, *β*_Θ_, *β*_*c*_, *c*_*p*_, *Q*, *D* and *R* are the Casson parameter, kinematic viscosity, electrical conductivity, magnetic parameter, the density of the fluid, parameter of porosity, thermal conductivity, thermal expansion coefficient, gravitational acceleration, temperature expansion coefficient, concentration expansion coefficient, specific heat at constant temperature, heat generating term, mass diffusivity and chemical reaction parameter respectively. *h*_*s*_ is the coefficient of convective heat transfer. The parameter *ε* = 0 when the magnetic field if fixed relative to the fluid (MFFRF) and *ε* = 1 when the magnetic field is fixed relative to the plate (MFFRP).

Introducing the following dimensionless variables, functions, and parameters

11 $$\begin{array}{l}\upsilon =\frac{u}{U},\,\zeta =\frac{U}{\nu }y,\,\tau =\frac{{U}^{2}}{\nu }t,\,\theta =\frac{{\rm{\Theta }}-{{\rm{\Theta }}}_{\infty }}{{{\rm{\Theta }}}_{\infty }},\,{\rm{\Phi }}=\frac{C-{C}_{\infty }}{{C}_{w}-{C}_{\infty }},\,\Re =\frac{\nu }{{U}^{2}}R,\\ f(\tau )=(\frac{\nu }{{U}^{2}}\tau ),\,\lambda =\frac{{h}_{s}\nu }{U},\,Sc=\frac{\nu }{D},\,\Pr =\frac{\mu {c}_{p}}{{k}_{1}},\,\delta =\frac{Q\nu }{{U}^{2}{k}_{1}},\,N=\frac{g{\beta }_{c}\nu ({C}_{w}-{C}_{\infty })}{{U}^{3}},\\ M=\frac{\sigma \nu {B}_{0}^{2}}{\rho {U}^{2}},\,\frac{1}{k}=\frac{\phi {\nu }^{2}}{{U}^{2}K},{N}_{0}=\frac{g{\beta }_{{\rm{\Theta }}}\nu {{\rm{\Theta }}}_{\infty }}{{U}^{3}},\end{array}$$

into equations (7–10) and choosing the characteristic velocity *U* to be equal to $\sqrt[3]{\nu g{\beta }_{{\rm{\Theta }}}{{\rm{\Theta }}}_{w}}$, we get the following dimensionless partial differential equations:

12 $$\begin{array}{rcl}\tfrac{\partial \upsilon (\zeta ,\tau )}{\partial \tau } & = & (1+\tfrac{1}{\beta })\tfrac{{\partial }^{2}\upsilon (\zeta ,\tau )}{\partial {\zeta }^{2}}-\tfrac{1}{K}(1+\tfrac{1}{\beta })\upsilon (\zeta ,\tau )-M(\upsilon (\zeta ,\tau )-\varepsilon f(\tau ))\\  &  & +{N}_{0}\theta (\zeta ,\tau )+N{\rm{\Phi }}(\zeta ,\tau );\zeta ,\tau  > 0,\end{array}$$

13 $${\rm{\Pr }}\frac{\partial \theta (\zeta ,\tau )}{\partial \tau }=\frac{{\partial }^{2}\theta (\zeta ,\tau )}{\partial {\zeta }^{2}}+{\rm{\Pr }}\,\delta \theta (\zeta ,\tau ),$$

14 $$\frac{\partial {\rm{\Phi }}(\zeta ,\tau )}{\partial \tau }=\frac{1}{Sc}\frac{{\partial }^{2}{\rm{\Phi }}(\zeta ,\tau )}{\partial {\zeta }^{2}}-\Re {\rm{\Phi }}(\zeta ,\tau ),$$

15 $$\begin{array}{l}\upsilon (\zeta ,\tau )=0,\,\theta (\zeta ,\tau )=0,\,{\rm{\Phi }}(\zeta ,\tau )=0;\,\zeta \ge 0,\,\tau =0\\ \upsilon (\zeta ,\tau )=f(\tau ),\,\frac{\partial \theta (\zeta ,\tau )}{\partial \zeta }=-\,\lambda (1+\theta ),\,{\rm{\Phi }}(\zeta ,\tau )=1;\,\tau \ge 0,\,\zeta =0\\ \upsilon (\zeta ,\tau )\to 0,\,\theta (\zeta ,\tau )\to 0,\,{\rm{\Phi }}(\zeta ,\tau )\to 0\,{\rm{as}}\,\zeta \to \infty \end{array}\}.$$

## The Solution of the Problem

The concentration and temperature fields corresponding to this problem are obtained easily from the previous work, here we are interested in the solution of the fluid velocity field. For this purpose we need to use the Laplace transform method for *θ*(*ζ*, *τ*), Φ(*ζ*, *τ*) and *υ*(*ζ*, *τ*).

By using the Laplace transform of Eqs (12–15), we developed the solutions:

16 $$\overline{\theta }(\zeta ,s)=\frac{1}{s}(\frac{{c}_{1}}{\sqrt{s-\delta }-\lambda })\,\exp \,(\,-\,\zeta \sqrt{{\rm{\Pr }}(s-\delta )}),$$

17 $$\overline{{\rm{\Phi }}}(\zeta ,s)=\frac{1}{s}\,\exp \,(\,-\,\zeta \sqrt{Sc(s-\Re )}),$$

18 $$\overline{\upsilon }(\zeta ,s)=F(s)\,\exp \,(\,-\,\zeta \sqrt{{c}_{2}(s+H)})+\,\frac{M\varepsilon F(s)}{s+H}\{1-\exp (\,-\,\zeta \sqrt{{c}_{2}(s+H)})\}+\,\frac{1}{s}\frac{1}{{c}_{3}(s-\frac{{c}_{4}}{{c}_{3}})}(\frac{{c}_{1}}{\sqrt{s-\delta }-\lambda })\{\exp \,(\,-\,\zeta \sqrt{{\rm{Re}}(s-\delta )})-\,\exp \,(\,-\,\zeta \sqrt{{c}_{2}(s+H)})\},\,\frac{1}{s}\frac{N}{{c}_{5}(s+\frac{{c}_{6}}{{c}_{5}})}\{\exp \,(\,-\,\zeta \sqrt{Sc(s-\Re )})-\,\exp \,(\,-\,\zeta \sqrt{{c}_{2}(s+H)})\}$$

where

$${c}_{1}=\frac{\lambda }{\sqrt{{\rm{\Pr }}}},\,{c}_{2}=\frac{\beta }{\beta +1},\,{c}_{3}={c}_{2}\,{\rm{\Pr }}-1,\,{c}_{4}={c}_{2}\,{\rm{\Pr }}\,\delta +H,\,{c}_{5}={c}_{2}Sc-1,\,{c}_{6}=-\,{c}_{2}Sc\Re -H.$$

Of course, *F*(*s*) and $\overline{\upsilon }(\zeta ,s)$ denoted the Laplace transform of *f*(*τ*) and *υ*(*ζ*,*τ*).

The transform boundary conditions are:

19 $$\begin{array}{l}\overline{\upsilon }(\zeta ,s)=F(s),\,\frac{\partial \overline{\theta }(\zeta ,s)}{\partial \zeta }=-\,\lambda (\frac{1}{s}+\overline{\theta }),\,\overline{{\rm{\Phi }}}(\zeta ,s)=\frac{1}{s};\,s\ge 0,\,\zeta =0\\ \overline{\upsilon }(\zeta ,s)\to 0,\,\overline{\theta }(\zeta ,s)\to 0,\,\overline{{\rm{\Phi }}}(\zeta ,s)\to 0\,{\rm{as}}\,\zeta \to \infty \end{array}\}.$$

Introducing the relations

$$\begin{array}{rcl}\frac{1}{s}\frac{1}{{c}_{3}(s-\frac{{c}_{4}}{{c}_{3}})}(\frac{{c}_{1}}{\sqrt{s-\delta }-\lambda }) & = & \frac{A}{s}+\frac{B}{s-\frac{{c}_{4}}{{c}_{3}}}+\frac{D}{s-\delta -{\lambda }^{2}}\\ \frac{1}{s}\frac{N}{{c}_{5}(s+\frac{{c}_{6}}{{c}_{5}})} & = & \frac{N}{{c}_{6}s}-\frac{N}{{c}_{6}(s+\frac{{c}_{6}}{{c}_{5}})}\end{array}$$

$$\begin{array}{l}A=\frac{{c}_{1}(\sqrt{\,-\,\delta }+\lambda )}{{c}_{4}{(\delta +{\lambda }^{2})}^{2}},B=\frac{{c}_{1}\sqrt{{c}_{3}}(\sqrt{{c}_{4}-{c}_{3}\delta }+\sqrt{{c}_{3}}\lambda )}{{c}_{4}-{c}_{3}\delta -{c}_{3}{\lambda }^{2}},\\ D=\frac{{c}_{1}}{(\delta +{\lambda }^{2})({c}_{3}\delta +{c}_{3}{\lambda }^{2}-{c}_{4})}\end{array}$$

into Eq. (18), by using the inverse Laplace method, the convolution theorem and Eqs (A1) and (A2) from the appendix, the velocity field is presented as:

20 $$\upsilon (\zeta ,\tau )={\upsilon }_{m}(\zeta ,\tau )+{\upsilon }_{{\rm{\Theta }}}(\zeta ,\tau )+{\upsilon }_{C}(\zeta ,\tau ),$$

where

21 $$\begin{array}{rcl}{\upsilon }_{m}(\zeta ,\tau ) & = & \frac{\zeta \sqrt{{c}_{2}}}{2\sqrt{\pi }}{\int }_{0}^{\tau }\,\frac{f(\tau -s)}{s\sqrt{s}}\,\exp \,(\,-\,\frac{{\zeta }^{2}{c}_{2}}{4s}-Hs)ds\\  &  & +\,\varepsilon M{\int }_{0}^{\tau }\,f(\tau -s)\,\exp \,(\,-\,Hs)erf(\frac{\zeta \sqrt{{c}_{2}}}{2\sqrt{s}})ds,\end{array}$$

22 $$\begin{array}{rcl}{\upsilon }_{{\rm{\Theta }}}(\zeta ,\tau ) & = & A[{\rm{\Psi }}(\zeta \sqrt{{\rm{Re}}},\tau ;-\,\delta ,0)-{\rm{\Psi }}(\zeta \sqrt{{c}_{2}},\tau ;H,0)]\\  &  & +\,B[{\rm{\Psi }}(\zeta \sqrt{{\rm{Re}}},\tau ;-\,\delta ,\frac{{c}_{4}}{{c}_{3}})-{\rm{\Psi }}(\zeta \sqrt{{c}_{2}},\tau ;H,\frac{{c}_{4}}{{c}_{3}})]\\  &  & +\,D[{\rm{\Psi }}(\zeta \sqrt{{\rm{Re}}},\tau ;-\,\delta ,\delta +{\lambda }^{2})-{\rm{\Psi }}(\zeta \sqrt{{c}_{2}},\tau ;H,\delta +{\lambda }^{2})],\end{array}$$

23 $$\begin{array}{rcl}{\upsilon }_{C}(\zeta ,\tau ) & = & \frac{N}{{c}_{6}}[{\rm{\Psi }}(\zeta \sqrt{Sc},\tau ;-\,\Re ,0)-{\rm{\Psi }}(\zeta \sqrt{{c}_{2}},\tau ;H,0)\\  &  & -\,{\rm{\Psi }}(\zeta \sqrt{Sc},\tau ;-\,\Re ,-\,\frac{{c}_{6}}{{c}_{5}})+{\rm{\Psi }}(\zeta \sqrt{{c}_{2}},\tau ;H,-\,\frac{{c}_{6}}{{c}_{5}})],\end{array}$$

where *υ*_*m*_(*ζ*, *τ*), *υ*_*C*_(*ζ*, *τ*) and *υ*_Θ_(*ζ*, *τ*) are the mechanical, concentration and thermal components of the velocity field respectively and the function Ψ(*ζ*, *τ*; *a*, *b*)is defined in appendix.

It is not difficult to show that the velocity field *υ*(*ζ*, *τ*), given by Eqs (19–23), satisfied the obligatory initial and boundary conditions. To verify the boundary condition (15), we rewrite *υ*(*ζ*, *τ*) in the equivalent form:

24 $$\begin{array}{rcl}{\upsilon }_{m}(\zeta ,\tau ) & = & \frac{2}{\sqrt{\pi }}\,{\int }_{\frac{\zeta \sqrt{{c}_{2}}}{2\sqrt{\tau }}}^{\infty }\,f(\tau -\frac{{\zeta }^{2}{c}_{2}}{4{s}^{2}})\,\exp \,(\,-\,\frac{H{\zeta }^{2}{c}_{2}}{4{s}^{2}}-{s}^{2})ds\\  &  & +\,\varepsilon M\,{\int }_{0}^{\tau }\,f(\tau -s)\,\exp \,(\,-\,Hs)\,erf\,(\frac{\zeta \sqrt{{c}_{2}}}{2\sqrt{s}})ds.\end{array}$$

As regards the limit of velocity at infinity, it results from that:

25 $$\mathop{\mathrm{lim}}\limits_{\zeta \to \,\infty }\upsilon (\zeta ,\tau )=\{\begin{array}{ll}0 & \varepsilon =0\\ M\,{\int }_{0}^{\tau }\,f(\tau -s)\,\exp \,(\,-\,Hs)ds & \varepsilon =1\end{array}.$$

In order to determine the skin fraction or shear on the plate, introducing Eq. (18) into

26 $${\tau }_{1}={-\frac{\partial \upsilon (\zeta ,t)}{\partial \zeta }|}_{\zeta =0}=-\,{{\rm{L}}}^{-1}\{{\frac{\partial \overline{\upsilon }(\zeta ,s)}{\partial \zeta }|}_{\zeta =0}\},$$

we find that (see also Eqs (A3–A5) from appendix)

27 $${\rm{T}}={{\rm{T}}}_{m}+{{\rm{T}}}_{{\rm{\Theta }}}+{{\rm{T}}}_{C},$$

where

28 $$\begin{array}{rcl}{{\rm{T}}}_{m} & = & {\int }_{0}^{\tau }\,f^{\prime} (\tau -s)\sqrt{{c}_{2}}[\sqrt{H}erf(\sqrt{Hs})+\frac{\exp (\,-\,Hs)}{\sqrt{\pi s}}]ds\\  &  & +\,\varepsilon \sqrt{H{c}_{2}}\,{\int }_{0}^{\tau }\,f^{\prime} (\tau -s)erf(\sqrt{Hs})ds,\end{array}$$

29 $$\begin{array}{rcl}{{\rm{T}}}_{{\rm{\Theta }}} & = & \sqrt{{c}_{2}}\{A\varphi (\tau ;H,0)+B\varphi (\tau ;H,-\,\frac{{c}_{4}}{{c}_{3}})+D\varphi (\tau ;H,-\,\delta -{\lambda }^{2})\}\\  &  & -\,\sqrt{{\rm{Re}}}\{A\varphi (\tau ;-\,\delta ,0)+B\varphi (\tau ;-\,\delta ,-\,\frac{{c}_{4}}{{c}_{3}})+D\varphi (\tau ;-\,\delta ,-\,\delta -{\lambda }^{2})\},\end{array}$$

30 $$\begin{array}{rcl}{{\rm{T}}}_{C} & = & \frac{N}{{c}_{6}}[\sqrt{{c}_{2}}\{\varphi (\tau ;H,0)-\varphi (\tau ;H,\frac{{c}_{6}}{{c}_{5}})\}\\  &  & -\,\sqrt{Sc}\{\varphi (\tau ;-\,\Re ,0)-\varphi (\tau ;-\,\Re ,\frac{{c}_{6}}{{c}_{5}})\}],\end{array}$$

are the mechanical, thermal and concentration components of the skin fraction and the function *φ*(*t*; *a*, *b*)is defined in the appendix.

## Case: 1 If *f*(*τ*) = *H*(*τ*)*τ*^*α*^ (Plate with Accelerated Motion)

It is clear from Eq. (15) that the concentration and thermal components of the velocity field be determined by the motion of the plate. The influence of mass and heat transfer is significant with some possible engineering applications. Taking, *f*(*τ*) = *H*(*τ*)*τ*^*α*^ using *α* > 0, the equations (20) and (27) become:

31 $$\begin{array}{rcl}{\upsilon }_{m}(\zeta ,\tau ) & = & \frac{\zeta \sqrt{{c}_{2}}}{2\sqrt{\pi }}\,{\int }_{0}^{\tau }\,\frac{{(\tau -s)}^{\alpha }}{s\sqrt{s}}\,\exp \,(\,-\,\frac{{\zeta }^{2}{c}_{2}}{4s}-Hs)ds\\  &  & +\,\varepsilon M\,{\int }_{0}^{\tau }\,{(\tau -s)}^{\alpha }\,\exp \,(\,-\,Hs)\,erf\,(\frac{\zeta \sqrt{{c}_{2}}}{2\sqrt{s}})ds\end{array}$$

32 $$\begin{array}{rcl}{{\rm{T}}}_{m} & = & {\int }_{0}^{\tau }\,\alpha {(\tau -s)}^{\alpha -1}\sqrt{{c}_{2}}[\sqrt{H}erf(\sqrt{Hs})+\frac{\exp (\,-\,Hs)}{\sqrt{\pi s}}]ds\\  &  & +\,\varepsilon \alpha \sqrt{H{c}_{2}}\,{\int }_{0}^{\tau }\,{(\tau -s)}^{\alpha -1}erf(\sqrt{Hs})ds,\end{array}$$

which relates to motions induced by a slow, constantly or highly accelerating plate.

When *α* = 0 the corresponding solution is:

33 $$\begin{array}{rcl}{\upsilon }_{0m}(\zeta ,\tau ) & = & \sqrt{{c}_{2}}H(\tau ){\rm{\Psi }}(\zeta \sqrt{{c}_{2}},\tau ;H,0)+\varepsilon H(\tau )\\  &  & \times \,[1-{\rm{\Psi }}(\zeta \sqrt{{c}_{2}},\tau ;H,0)-\exp \,(\,-\,Hs)\,erf\,(\frac{\zeta \sqrt{{c}_{2}}}{2\sqrt{s}})],\end{array}$$

34 $${{\rm{T}}}_{0m}=\sqrt{{c}_{2}}H(\tau )[\sqrt{H}erf(H\tau )+\frac{\exp (\,-\,H\tau )}{\sqrt{\pi \tau }}]-\varepsilon \sqrt{H{c}_{2}}H(\tau )erf(\sqrt{H\tau }).$$

More exactly when *α* = *n* (a natural number) can be written as:

35 $$\begin{array}{rcl}{\upsilon }_{nm}(\zeta ,\tau ) & = & {\int }_{0}^{\tau }\,{\int }_{0}^{{s}_{1}}\,{\int }_{0}^{{s}_{2}}\,\ldots \,{\int }_{0}^{{s}_{n-1}}\,{\upsilon }_{0m}(\zeta ,{s}_{n})d{s}_{1}d{s}_{2}\ldots d{s}_{n},\\ {{\rm{T}}}_{nm}(\zeta ,\tau ) & = & {\int }_{0}^{\tau }\,{\int }_{0}^{{s}_{1}}\,{\int }_{0}^{{s}_{2}}\,\ldots \,{\int }_{0}^{{s}_{n-1}}\,{{\rm{T}}}_{0m}(\zeta ,{s}_{n})d{s}_{1}d{s}_{2}\ldots d{s}_{n}.\end{array}$$

## Case: 2 If *f*(*τ*) = *H*(*τ*) cos(*ωτ*) *or H*(*τ*) sin(*ωτ*) (Plate with Oscillating Motion)

Introducing *f*(*τ*) = *H*(*τ*) cos(*ωτ*) *or* sin(*ωτ*) (corresponds to Stokes’ second problem) into Eqs (20) and (27) and using the fact that

$$H^{\prime} (\tau )=\delta (\tau )\,{\rm{and}}\,{\int }_{0}^{\tau }\,\delta (\tau -s)f(s)ds={\int }_{0}^{\tau }\,\delta (s)f(\tau -s)ds=f(\tau ),$$

where *δ*(.) is the Direct delta function, we get

36 $$\begin{array}{rcl}{\upsilon }_{cm}(\zeta ,\tau ) & = & \frac{\zeta \sqrt{{c}_{2}}}{2\sqrt{\pi }}\,{\int }_{0}^{\tau }\,\frac{\cos \,[\omega (\tau -s)]}{s\sqrt{s}}\,\exp \,(\,-\,\frac{{\zeta }^{2}{c}_{2}}{4s}-Hs)ds\\  &  & +\,\varepsilon H{\int }_{0}^{\tau }\,\cos \,[\omega (\tau -s)]\,\exp \,(\,-\,Hs)erf(\frac{\zeta \sqrt{{c}_{2}}}{2\sqrt{s}})ds,\end{array}$$

37 $$\begin{array}{rcl}{\upsilon }_{sm}(\zeta ,\tau ) & = & \frac{\zeta \sqrt{{c}_{2}}}{2\sqrt{\pi }}\,{\int }_{0}^{\tau }\,\frac{\sin \,[\omega (\tau -s)]}{s\sqrt{s}}\,\exp \,(\,-\,\frac{{\zeta }^{2}{c}_{2}}{4s}-Hs)ds\\  &  & +\,\varepsilon H\,{\int }_{0}^{\tau }\,\sin \,[\omega (\tau -s)]\,\exp \,(\,-\,Hs)erf(\frac{\zeta \sqrt{{c}_{2}}}{2\sqrt{s}})ds,\end{array}$$

38 $$\begin{array}{rcl}{{\rm{T}}}_{cm} & = & H(\tau )\{\sqrt{{c}_{2}}[\sqrt{H}erf(\sqrt{Hs})+\frac{\exp (\,-\,Hs)}{\sqrt{\pi s}}+\varepsilon \sqrt{H}erf(\sqrt{Hs})]\}\\  &  & -\,\omega {\int }_{0}^{\tau }\,\sin [\omega (\tau -s)]\sqrt{{c}_{2}}[\sqrt{H}erf(\sqrt{Hs})+\frac{\exp (\,-\,Hs)}{\sqrt{\pi s}}]ds\\  &  & -\,\varepsilon \omega \sqrt{H{c}_{2}}{\int }_{0}^{\tau }\,\sin [\omega (\tau -s)]erf(\sqrt{Hs})ds,\end{array}$$

39 $$\begin{array}{rcl}{{\rm{T}}}_{sm} & = & \omega \,{\int }_{0}^{\tau }\,\cos [\omega (\tau -s)]\sqrt{{c}_{2}}[\sqrt{H}erf(\sqrt{Hs})+\frac{\exp (\,-\,Hs)}{\sqrt{\pi s}}]ds\\  &  & +\,\varepsilon \omega \sqrt{H{c}_{2}}{\int }_{0}^{\tau }\,\cos [\omega (\tau -s)]erf(\sqrt{Hs})ds.\end{array}$$

The dimensional velocities for sine and cosine oscillations describe the fluid motion for unsteady state solution. After that the transients disappear, they reduce to steady state (permanent) solutions:

40 $$\begin{array}{rcl}{\upsilon }_{cmp}(\zeta ,\tau ) & = & \frac{\zeta \sqrt{{c}_{2}}}{2\sqrt{\pi }}{\int }_{0}^{\infty }\,\frac{\cos [\omega (\tau -s)]}{s\sqrt{s}}\,\exp \,(\,-\,\frac{{\zeta }^{2}{c}_{2}}{4s}-Hs)ds\\  &  & +\,\varepsilon H\,{\int }_{0}^{\infty }\,\cos [\omega (\tau -s)]\,\exp \,(\,-\,Hs)erf(\frac{\zeta \sqrt{{c}_{2}}}{2\sqrt{s}})ds\end{array}$$

41 $$\begin{array}{rcl}{\upsilon }_{smp}(\zeta ,\tau ) & = & \frac{\zeta \sqrt{{c}_{2}}}{2\sqrt{\pi }}\,{\int }_{0}^{\infty }\,\frac{\sin [\omega (\tau -s)]}{s\sqrt{s}}\,\exp \,(\,-\,\frac{{\zeta }^{2}{c}_{2}}{4s}-Hs)ds\\  &  & +\,\varepsilon H\,{\int }_{0}^{\infty }\,\sin [\omega (\tau -s)]\,\exp \,(\,-\,Hs)erf(\frac{\zeta \sqrt{{c}_{2}}}{2\sqrt{s}})ds,\end{array}$$

which are independent of initial condition and periodic in time. Though, they satisfy the boundary condition and the governing equation.

Equations (40) and (41) Can be written using simple but beautiful forms by using Eqs (A8) and (A9) from appendix,

42 $$\begin{array}{rcl}{\upsilon }_{cmp}(\zeta ,\tau ) & = & \exp (\,-\,m\zeta )\,\cos \,(\omega \tau -{n}_{1}\zeta )\\  &  & +\,\tfrac{\varepsilon H}{\sqrt{{H}^{2}+{\omega }^{2}}}\{\,\cos (\omega \tau -\phi )-\exp (\,-\,m\zeta )\,\cos \,(\omega \tau -{n}_{1}\zeta -\phi )\},\end{array}$$

43 $$\begin{array}{rcl}{\upsilon }_{smp}(\zeta ,\tau ) & = & \exp (\,-\,m\zeta )\,\sin \,(\omega \tau -{n}_{1}\zeta )\\  &  & +\,\tfrac{\varepsilon H}{\sqrt{{H}^{2}+{\omega }^{2}}}\{\,\sin \,(\omega \tau -\phi )-\exp \,(\,-\,m\zeta )\,\sin \,(\omega \tau -{n}_{1}\zeta -\phi )\},\end{array}$$

where $$m=\sqrt{\frac{\sqrt{{H}^{2}+{\omega }^{2}}+H}{2}}$$, $${n}_{1}=\sqrt{\frac{\sqrt{{H}^{2}+{\omega }^{2}}-H}{2}}$$ and $$\phi =arctg(\frac{\omega }{H})$$.

Taking the limit *ζ* → ∞, the Eqs (42) and (43) become

44 $${\upsilon }_{cmp}(\infty ,\tau )=\{\begin{array}{ll}0 & if\,\varepsilon =0\\ \frac{H}{\sqrt{{H}^{2}+{\omega }^{2}}}\,\cos \,(\omega \tau -\phi ) & if\,\varepsilon =1\end{array}$$

45 $${\upsilon }_{smp}(\infty ,\tau )=\{\begin{array}{ll}0 & if\,\varepsilon =0\\ \frac{H}{\sqrt{{H}^{2}+{\omega }^{2}}}\,\sin \,(\omega \tau -\phi ) & if\,\varepsilon =1\end{array}.$$

## Results and Discussion

Exact solution of MHD natural convection radiative flow of Casson fluid over a moving vertical plate with chemical reaction, concentration, and Newtonian heating are developed by using the Laplace transform method. The magnetic field is taken relative i.e. fixed to the plate or to the fluid. The effect of numerous physical parameters on velocity *υ*(*ζ*, *τ*), temperature *θ*(*ζ*, *τ*) and concentration Φ(*ζ*, *τ*) profiles have schemed in Figs 1, 2, 3, 4, 5, 6, 7, 8, 9, 10, 11, 12, 13, 14 and 15.

**Figure 1:**
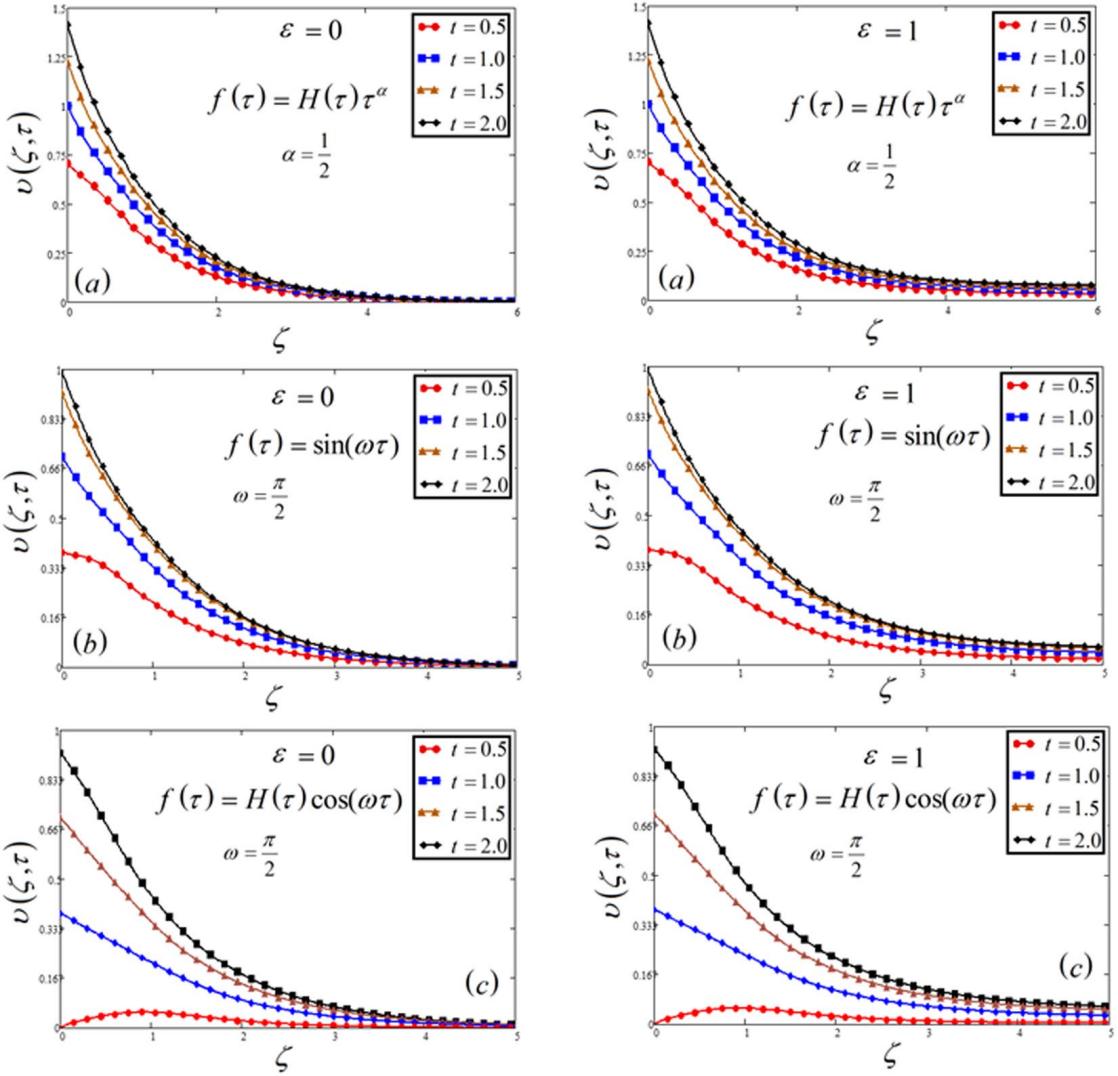
Velocity plot profile against time *t* for the variable and oscillating plate.

**Figure 2:**
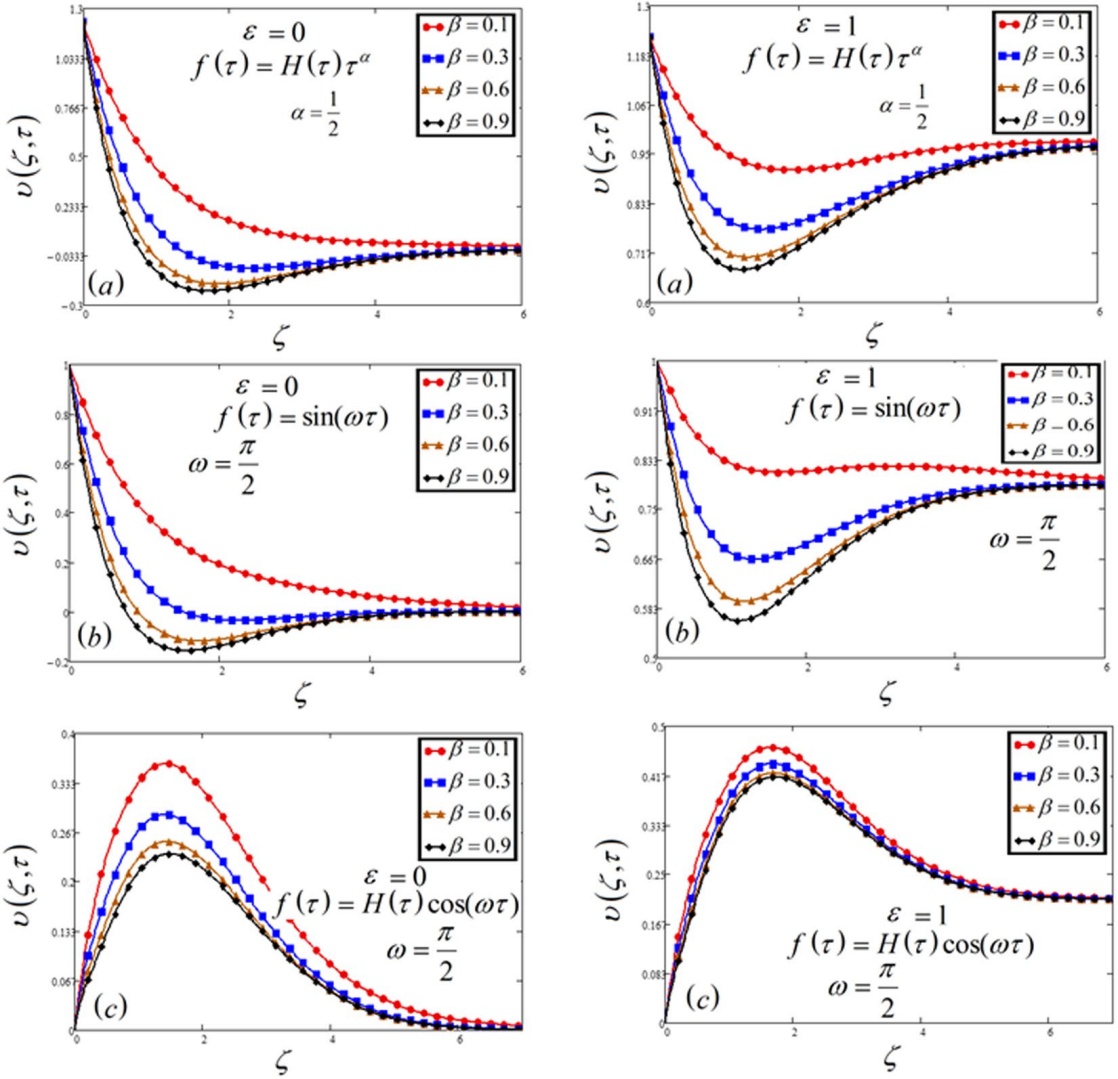
Velocity plot against Casson parameter *β* for the variable and oscillating plate.

**Figure 3:**
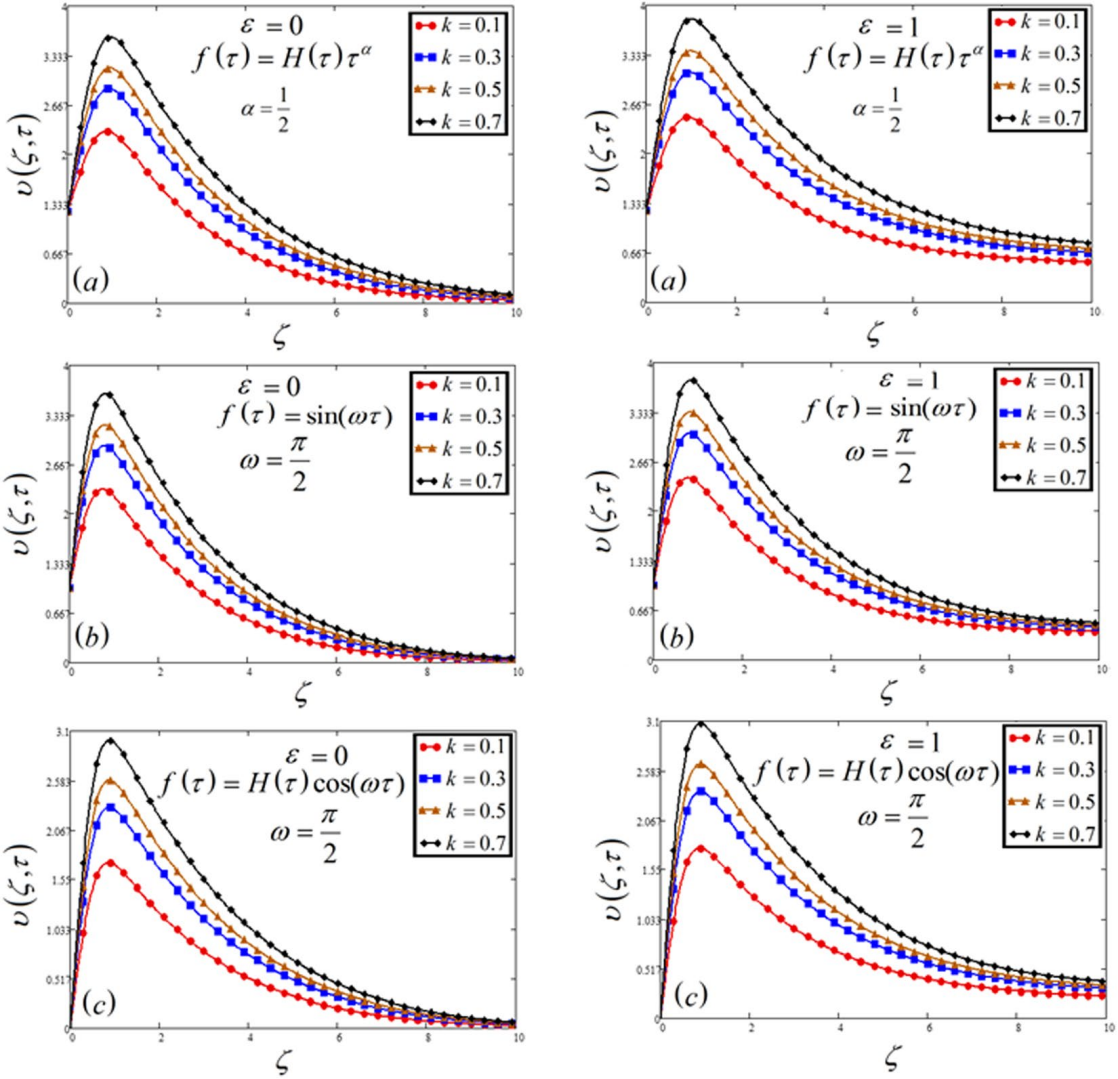
Velocity plot against permeability of porous medium *k* for the variable and oscillating plate.

**Figure 4:**
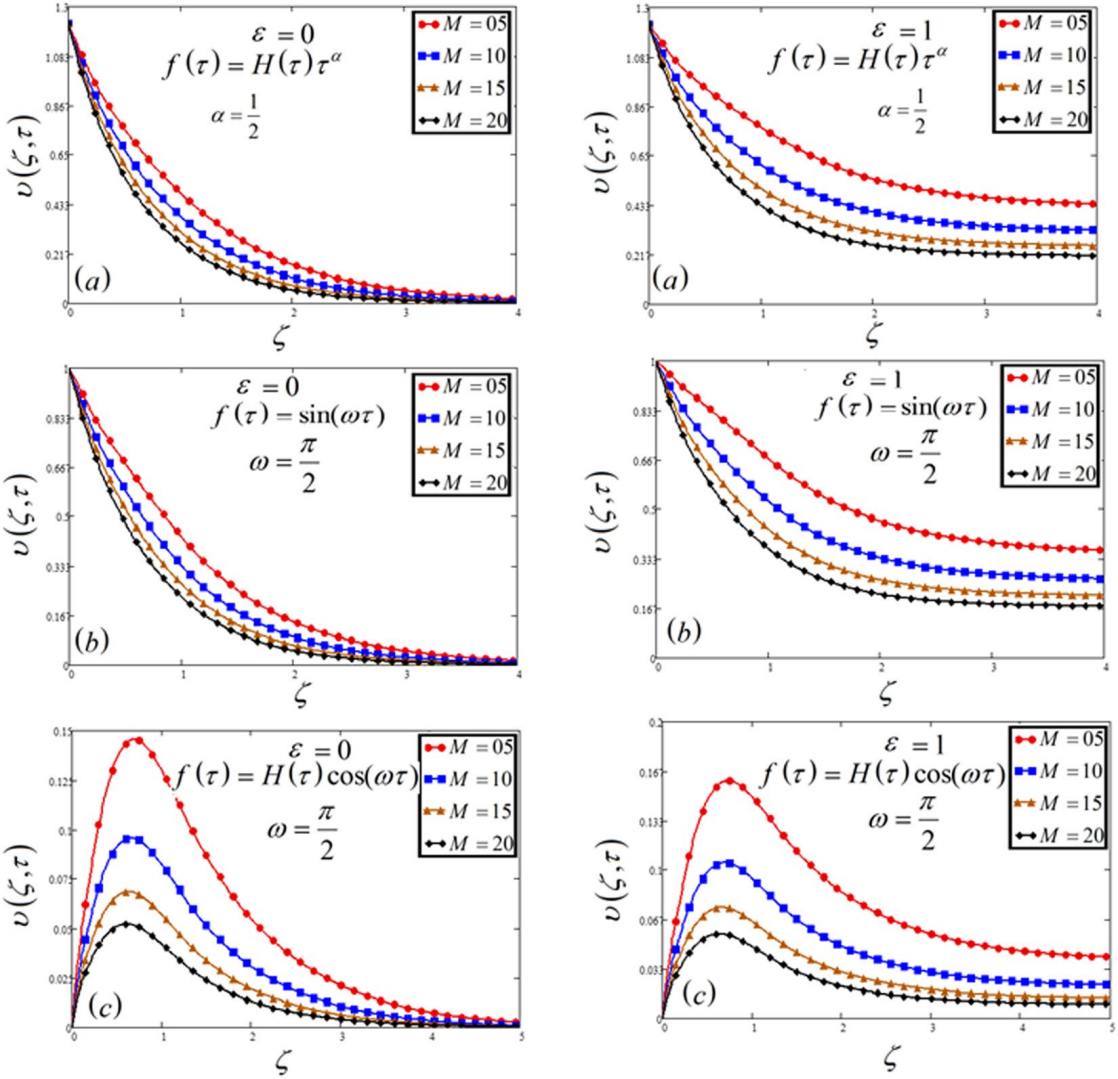
Velocity plot against magnetic parameter *M* for the variable and oscillating plate.

**Figure 5:**
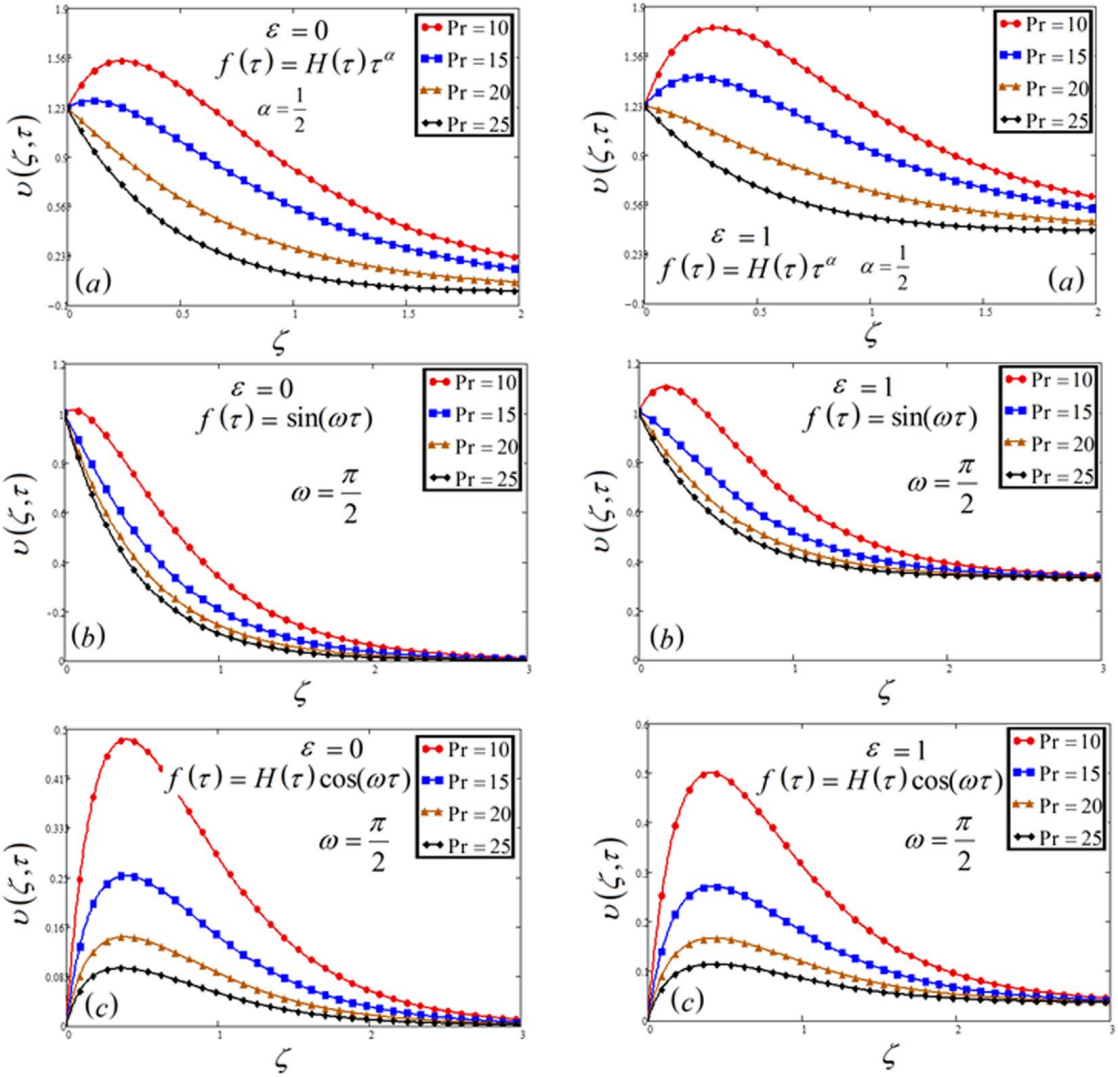
Velocity plot against Prandtl number Pr for the variable and oscillating plate.

**Figure 6:**
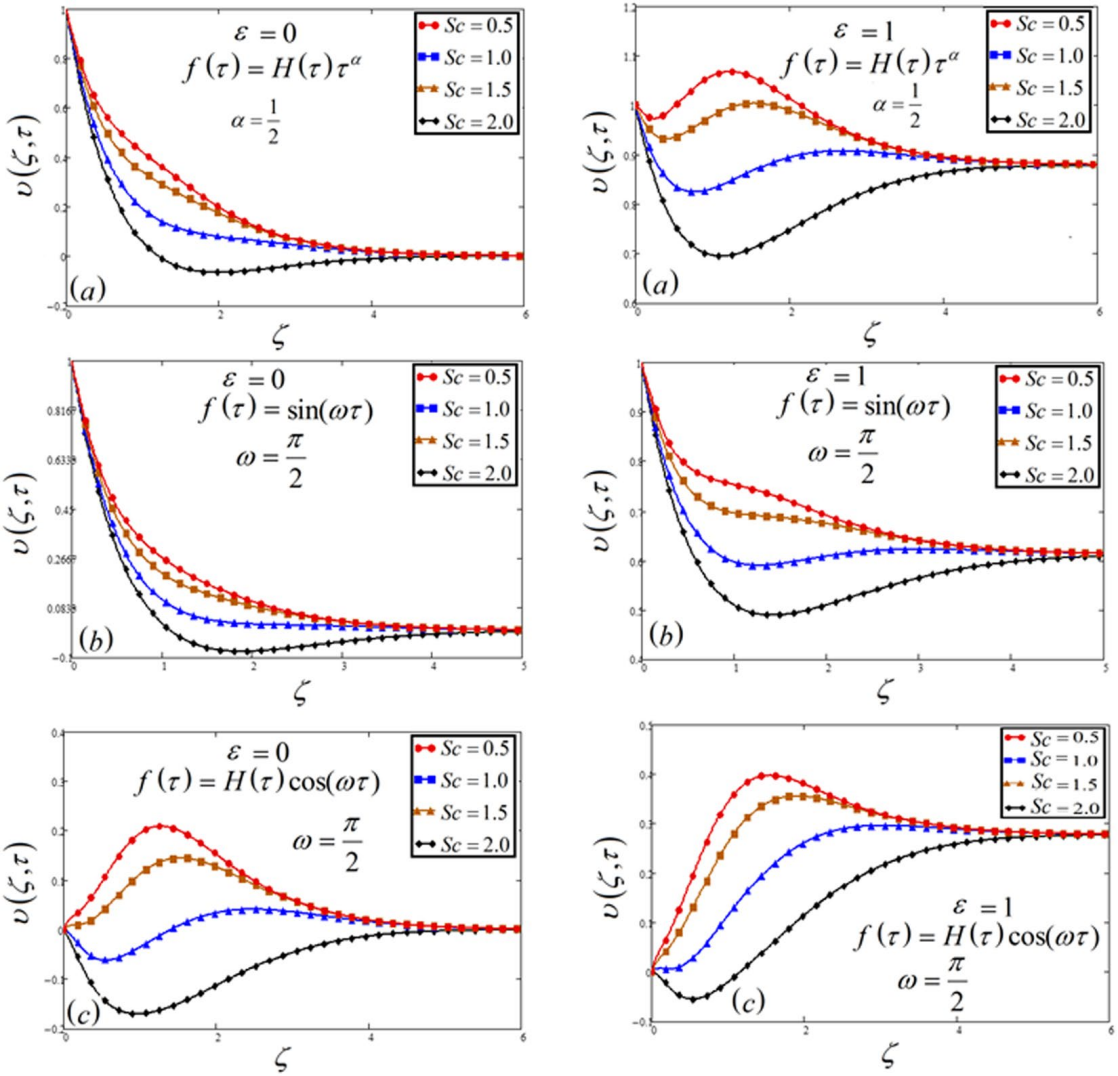
Velocity plot against Schmidt number *Sc* for the variable and oscillating plate.

**Figure 7:**
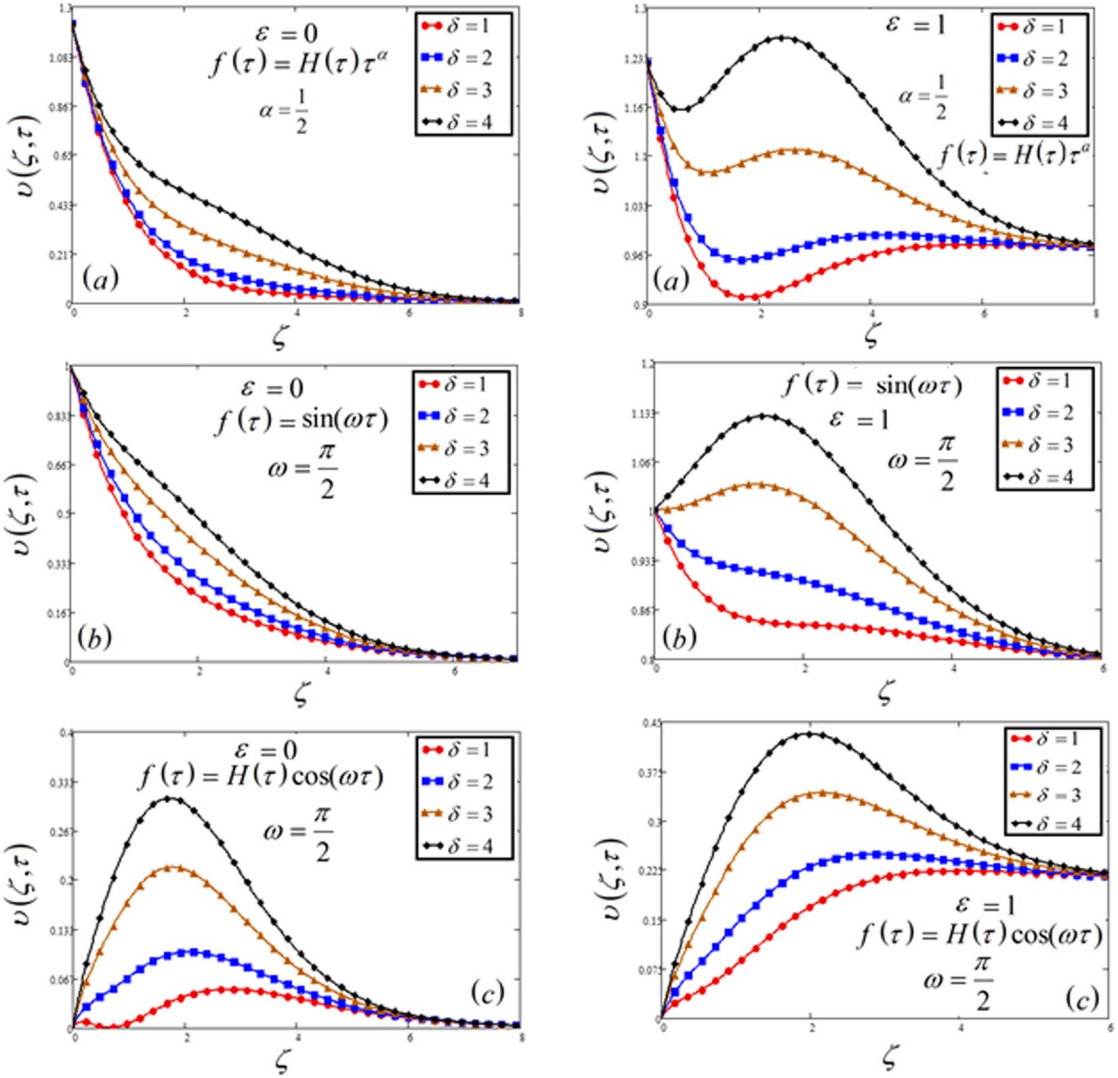
Velocity plot against heat generation parameter *δ* for the variable and oscillating plate.

**Figure 8:**
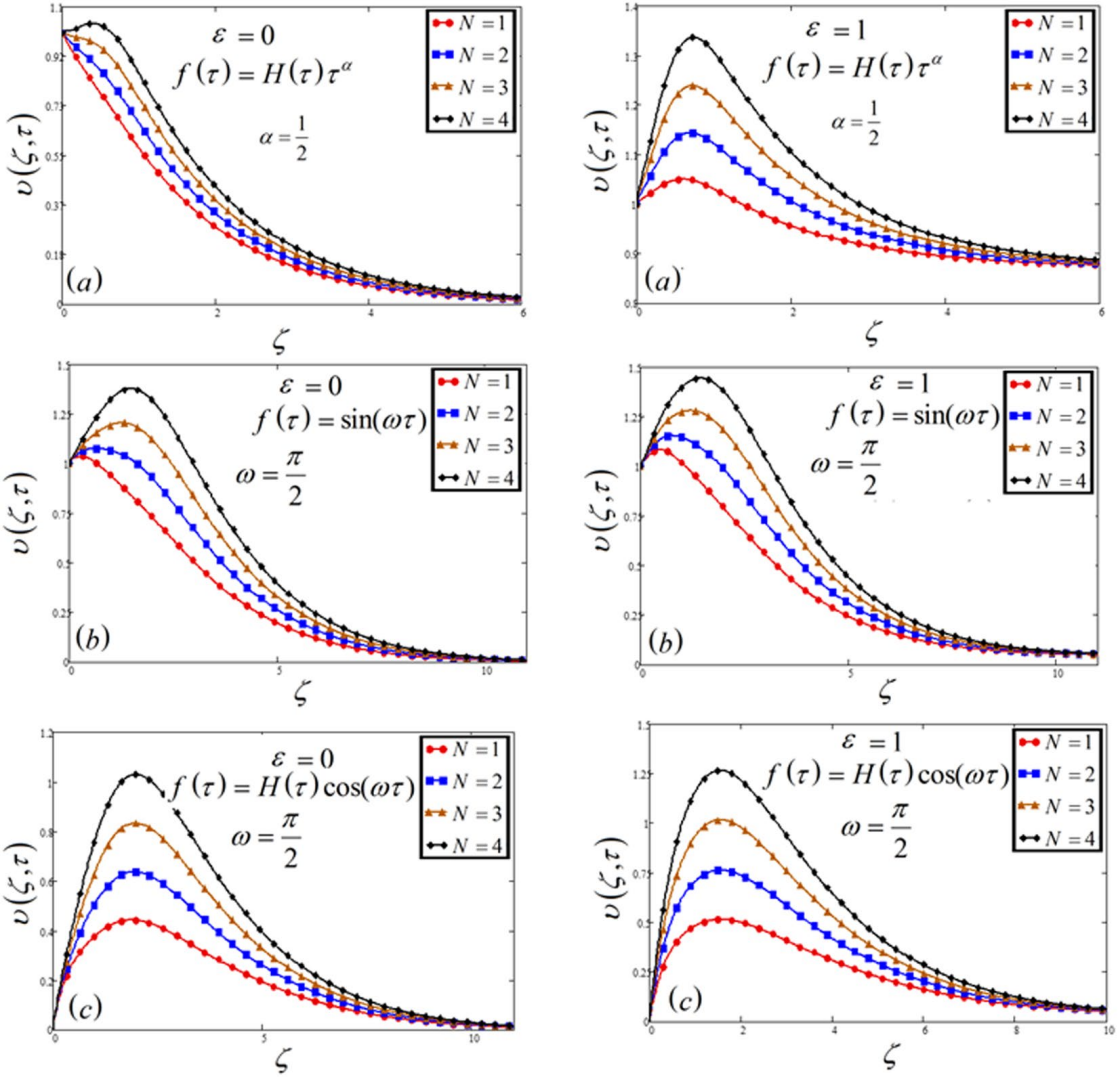
Velocity plot against buoyancy force parameter *N* for the variable and oscillating plate.

**Figure 9:**
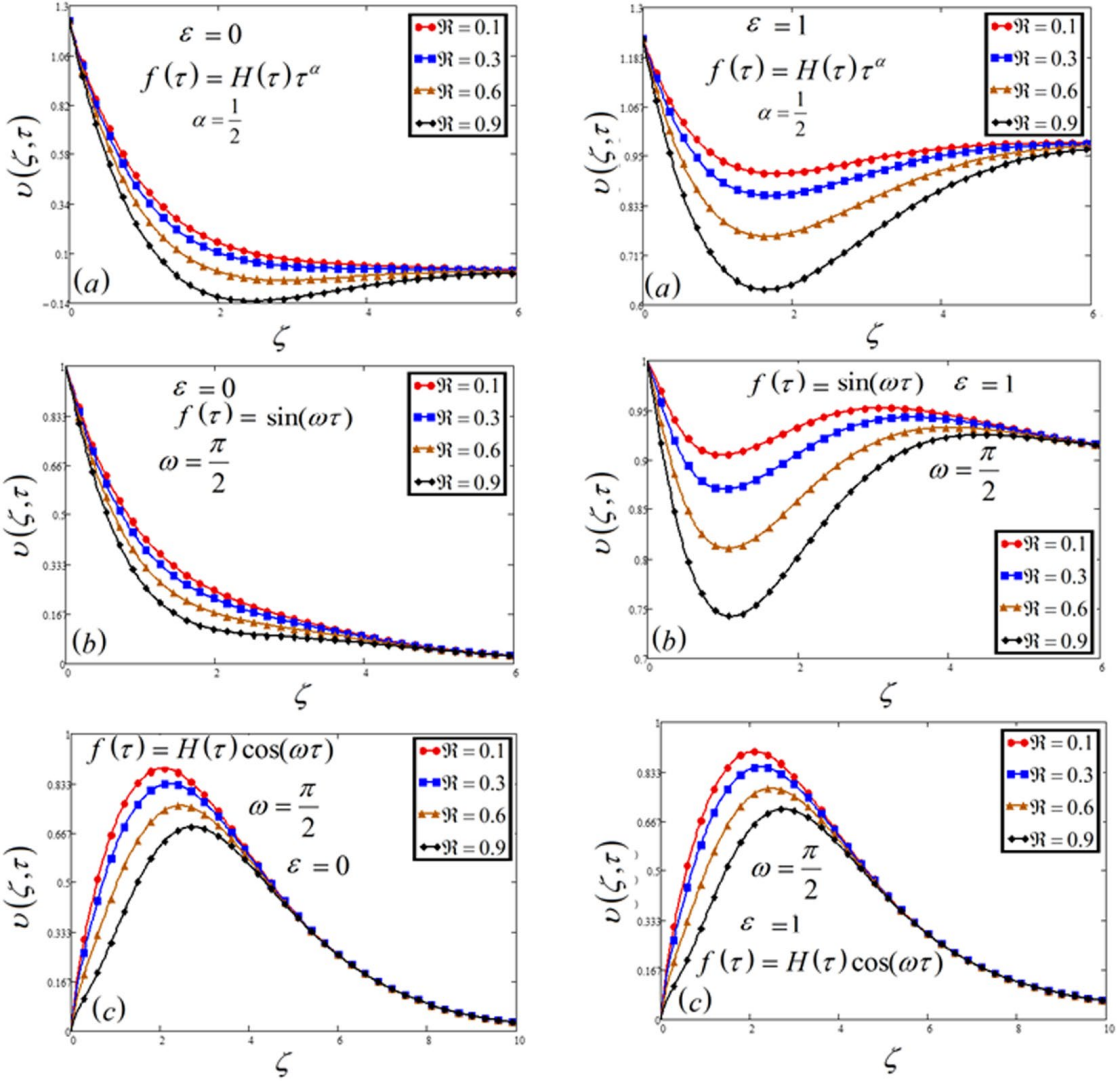
Velocity plot against chemical reaction parameter $\Re$ for the variable and oscillating plate.

**Figure 10:**
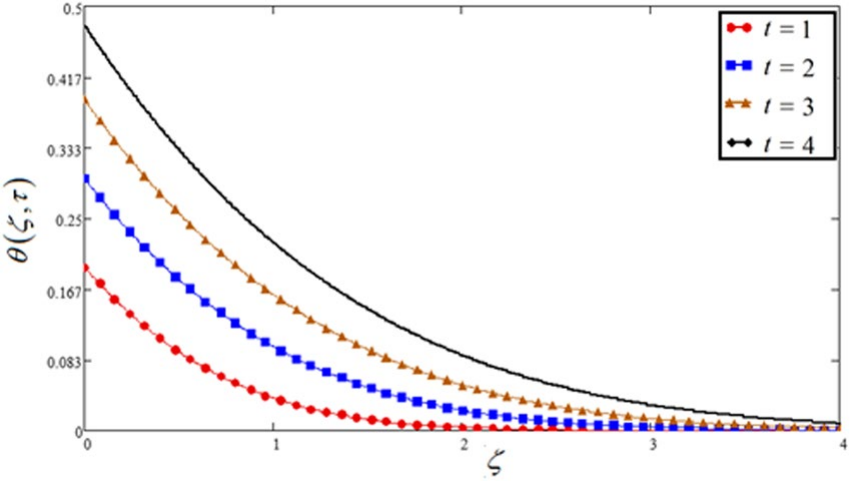
Temperature plot against time *t*.

**Figure 11:**
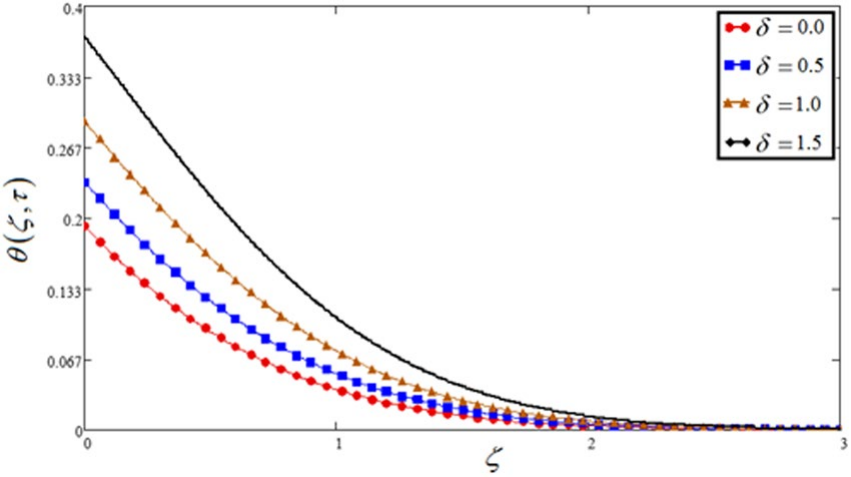
Temperature plot against heat generation parameter *δ*.

**Figure 12:**
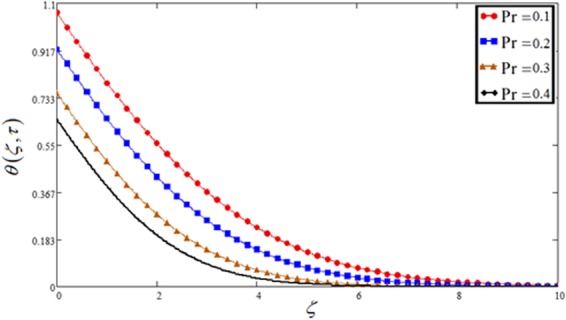
Temperature plot against Prandtl number Pr.

**Figure 13:**
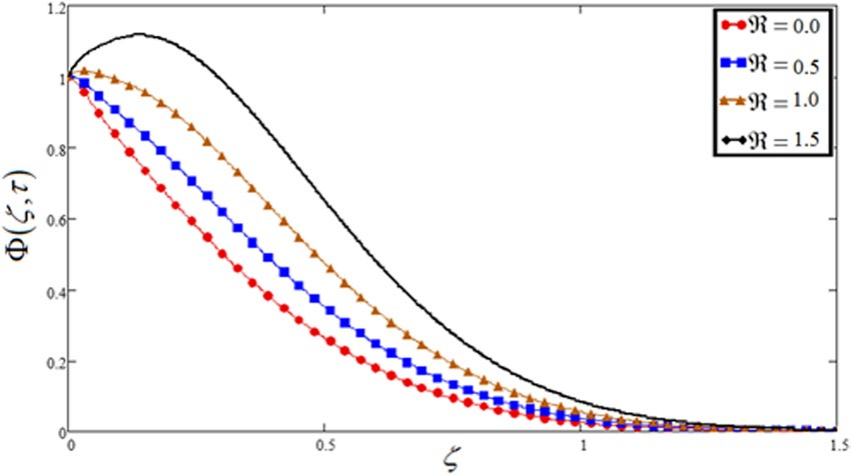
Concentration plot against chemical reaction $\Re$.

**Figure 14:**
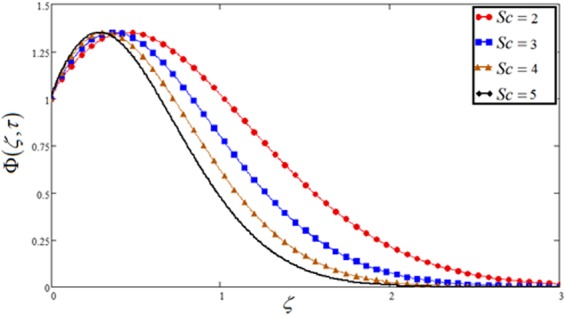
Concentration plot against Schmidt number *Sc*.

**Figure 15:**
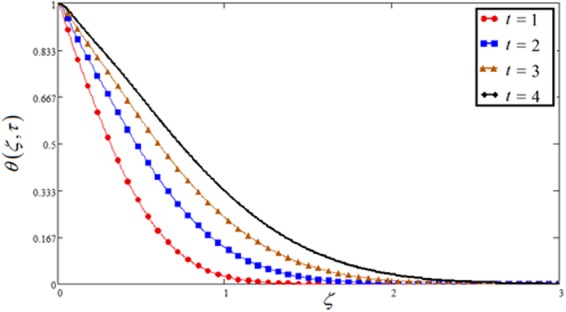
Concentration plot against time *t*.

Figures 1, 10 and 15 show the influence of time *t* on concentration, temperature and velocity profile. In all three Figs, time *t* shows an increasing effects on all the three profiles. Moreover, Fig. 1 has included all the three cases of variable, sine and cosine oscillating plate conditions. An identical behavior is noticed for all the three cases. It is clear that for increasing time, MFFRP has greater velocity than MFFRF.

The influence of *β* on the velocity profile is plotted in Fig. 2. The maximum value of *β* leads to decrease the velocity as well as the boundary layer thickness. It is because increasing *β* reduced the boundary layer thickness. All the three cases have the same effects.

The influence of permeability of the porous medium *k* on velocity profile, for a variable and oscillating (sine and cosine) plate is highlighted in Fig. 3. It is clear that for decreasing of velocity, we need to decrease the permeability of a porous medium. It is indeed due to decreasing drag force, which leads to the decreasing of velocity. In other words, the resistance of the porous medium is depressed which increases the momentum development of the flow regime, ultimately accelerate the velocity of the fluid.

Magnetic parameter *M* leads to a decrease in the velocity profile as shown in Fig. 4. It is due to Lorentz force. Lorentz force works in the opposite direction of the fluid which makes a decreasing effect on velocity. Furthermore, increasing value of *M* increases resistive forces which resist the fluid flow and thus decreasing the fluid velocity.

The impact of Pr on velocity as well as temperature profile is discussed in Figs 5 and 12. For both profiles, the increase of Pr leads to falling in velocity and temperature. The low rate of thermal diffusion causes arising in velocity boundary layer thickness. Pr controls the relative thickness of the thermal and momentum boundary layers in the problems of heat transfer. Consequently Pr can be utilized to expand the percentage of cooling.

Figures 6 and 14 illustrate variation in velocity as well as concentration respectively for changed values of Schmidt number *Sc*. Growth in Schmidt number declines the concentration boundary layer thickness. As Schmidt number is the ratio between viscous forces and mass diffusivity, therefore, it reduced the consecration as well as velocity profile. Physically, if the increase occurs in viscous forces, it leads to a decrease in the velocity of the fluid.

Figures 7 and 11 highlight the effect of velocity and concentration respectively against heat generation parameter *δ*. This shows the enhancement of temperature profiles by increasing heat generation parameter, which is because of a thickness of the thermal boundary layer. Physically, the kinetic energy of the fluid particles increases if increase the *δ*, so temperature and thickness of the boundary layer increase, which leads to an increase in velocity.

The variation of the ratio of buoyancy force *N* is highlighted in Fig. 8. This shows the increasing effect on the velocity profile. Increase in *N* means, the increase of concentration gradients which leads the significant of buoyancy forces and hence observed a small rising near the plate.

Figures 9 and 13 highlight the effect of the parameter of chemical reaction $\Re$ on velocity and concentration profiles. The increasing behavior of $\Re$ reduced the concentration and velocity profiles. Physically, it is true because higher values of v make fall in the chemical molecular diffusivity, i.e., less diffusion. An increase in $\Re$ will suppress species concentration. The concentration distribution decreases at all points of the flow field with the increase in $\Re$.

In Figs 1, 2, 3, 4, 5, 6, 7, 8 and 9, both cases that is MFFRF (*ε* = 0) and MFFRP (*ε* = 1) are analyzed. It is observed that MFFRP have greater velocity profile as compare to MFFRF, and the same result is analyzed for all three cases which is plotted in (a), (b) and (c) for variable and oscillating (sine and cosine oscillations) conditions.

**Figure 16:**
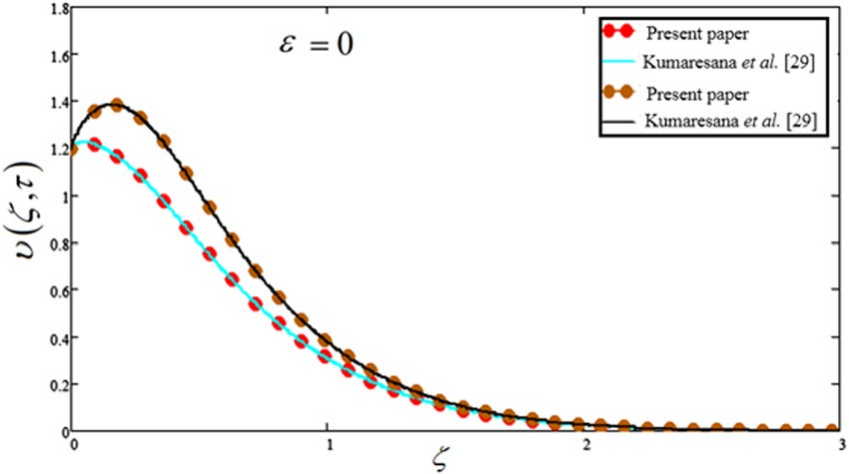
Comparison plot of the present solution with Kumaresana *et al*.^29^.

The comparison of present solution is made in Fig. 16 with published result of Kumaresan *et al*.^29^. It is observed that in the absence of Casson, Newtonian heating and chemical reaction parameters i.e. $\beta =\lambda =\Re =0$, the result is found identical to the solution obtained in^29^ [see Fig. 11]. This shows the validity of our obtained solutions.

## Conclusion

An exact analysis of unsteady, incompressible, Casson fluid of free convection flow ended moving vertical plate set in a pours medium, the constant magnetic field is vertically applied to the plate with the MFFRF and MFFRP, and our interest to focus on the motion of the fluid. The influence of temperature and concentration is considered with the presence of thermal radiation and Newtonian-heating. The method of Laplace transformation is used to determine the closed form of concentration, velocity and temperature and the corresponding skin fraction are established in the term of error and complementary error functions. The velocity is obtained for a variable and oscillating plate. Both the velocity and skin fraction are presented in the sum of their mechanical, thermal and concentration components. For comparison, the concentration, velocity and temperature profile have schemed graphically and the effect of the various parameter is studied. The main findings are as follows:

- Time *t* have a similar effect on velocity, temperature and concentration profile.
- *k*, *δ*, *N* have increasing effect on velocity while *β*, *M*, Pr, *Sc*, $\Re$ have a decreasing effect on velocity.
- The comparison study of MFFRP and MFFRF is highlighted and it saw that the velocity of MFFRP is greater than MFFRF.
- Contributions of concentration, thermal and mechanical component of velocity and skin fraction on the motion of fluid are significant and they cannot be ignored.
- The velocity profile is compared for variable and oscillating plate motion.

**Appendix**

A1 $$\begin{array}{rcl}{L}^{-1}\{{e}^{-y\sqrt{q}}\} & = & \frac{y}{2t\sqrt{\pi t}}{e}^{-\frac{{y}^{2}}{4t}},\\ {L}^{-1}\{\frac{{e}^{-y\sqrt{q}}}{q}\} & = & erfc(\frac{y}{2\sqrt{t}}),\\ {L}^{-1}\{\frac{{e}^{-y\sqrt{q+a}}}{q-b}\} & = & \psi (y,t;a,b).\end{array}$$

A2 $$\begin{array}{rcl}\psi (y,t;a,b) & = & \frac{{e}^{bt}}{2}[{e}^{-y\sqrt{a+b}}erfc(\frac{y}{2\sqrt{t}}-\sqrt{(a+b)t})\\  &  & +\,{e}^{y\sqrt{a+b}}erfc(\frac{y}{2\sqrt{t}}+\sqrt{(a+b)t})].\end{array}$$

A3 $${L}^{-1}\{qF(q)\}=f^{\prime} (t)+\delta (t)f(0)\,if\,{L}^{-1}\{F(q)\}=f(t)\delta (\cdot )\,{\rm{is}}\,{\rm{the}}\,{\rm{direct}}\,{\rm{delta}}\,{\rm{function}}$$

A4 $${L}^{-1}\{\frac{1}{(q+b)\sqrt{q+a}}\}=\frac{{e}^{bt}}{\sqrt{q-b}}erfc(\sqrt{(q-b)t}),\,{L}^{-1}\{\frac{1}{\sqrt{q}}\}=\frac{1}{\sqrt{\pi t}}.$$

A5 $${L}^{-1}\{\frac{\sqrt{q+a}}{q+b}\}=\frac{{e}^{-at}}{\sqrt{\pi t}}+\frac{{e}^{-bt}}{\sqrt{q-b}}erf(\sqrt{(q-b)t})=\varphi (t;a,b).$$

A6 $$\begin{array}{rcl}{\int }_{0}^{t}\,\frac{1}{\sqrt{q}}\,\exp \,(\,-\,\frac{{y}^{2}}{4q}-aq)dq & = & \frac{\sqrt{\pi }}{2\sqrt{a}}\{{e}^{-y\sqrt{a}}erfc(\frac{y}{2\sqrt{t}}-\sqrt{at})\\  &  & -\,{e}^{y\sqrt{a}}erfc(\frac{y}{2\sqrt{t}}+\sqrt{at})\}.\end{array}$$

A7 $$\begin{array}{rcl}{\int }_{0}^{t}\frac{1}{q\sqrt{q}}\,\exp \,(\,-\,\frac{{y}^{2}}{4q}-aq)dq & = & \frac{\sqrt{\pi }}{y}\{{e}^{-y\sqrt{a}}erfc(\frac{y}{2\sqrt{t}}-\sqrt{at})\\  &  & -\,{e}^{y\sqrt{a}}erfc(\frac{y}{2\sqrt{t}}+\sqrt{at})\}.\end{array}$$

A8 $${\int }_{0}^{\infty }\,{e}^{-{p}^{2}{s}^{2}-\frac{{q}^{2}}{{s}^{2}}}\,\cos \,({a}^{2}{s}^{2}+\frac{{b}^{2}}{{s}^{2}})ds=\tfrac{\sqrt{\pi }}{{2}^{4}\sqrt{{p}^{4}+{a}^{4}}}{e}^{-2c\cos (\alpha +\beta )}\,\cos \,[\alpha +2c\,\sin \,(\alpha +\beta )].$$

A9 $$\begin{array}{l}{\int }_{0}^{\infty }\,{e}^{-{p}^{2}{s}^{2}-\frac{{q}^{2}}{{s}^{2}}}\,\sin \,({a}^{2}{s}^{2}+\frac{{b}^{2}}{{s}^{2}})ds=\frac{\sqrt{\pi }}{{2}^{4}\sqrt{{p}^{4}+{a}^{4}}}{e}^{-2c\cos (\alpha +\beta )}\,\sin \,[\alpha +2c\,\sin \,(\alpha +\beta )].\\ where\,\alpha =\frac{1}{2}arctg(\frac{{a}^{2}}{{p}^{2}}),\,\beta =\frac{1}{2}arctg(\frac{{b}^{2}}{{q}^{2}}),\,and\,c=\sqrt[4]{({p}^{4}+{a}^{4})({q}^{4}+{b}^{4})}.\end{array}$$

## Electronic supplementary material

Supl infomarion file
