# Supplementary material for: Effects of Relative Magnetic Field, Chemical Reaction, Heat Generation and Newtonian Heating on Convection Flow of Casson Fluid over a Moving Vertical Plate Embedded in a Porous Medium
Source: Sci Rep. 2019 Jan 23;9:400. doi: 10.1038/s41598-018-36243-0 (PMC6344514; doi:10.1038/s41598-018-36243-0)
Supplement: Supplementary file 1 — Supl infomarion file [file 41598_2018_36243_MOESM1_ESM.docx]

**Effects of Relative Magnetic Field, Chemical Reaction, Heat Generation and Newtonian Heating on Convection Flow of Casson Fluid over a Moving Vertical Plate Embedded in a Porous Medium**

Dolat Khan^2^, Arshad Khan^3,^ , Ilyas Khan^1*^, Farhad Ali^2,4^ _,_ Faizan ul Karim^2^ and I. Tlili^5^

*^1^Faculty of Mathematics and Statistics, Ton Duc Thang University, Ho Chi Minh City, Vietnam.*

*^2^Department of Mathematics, City University of Science and Information Technology,*

*Peshawar, 25000, Pakistan.*

*^3^Institute of Business and Management Sciences, The University of Agriculture, Peshawar, Khyber Pakhtunkhwa Pakistan.*

*^4^Computational Analysis Research Group, Ton Duc Thang University, Ho Chi Minh City, Vietnam.*

*^5^Energy and Thermal Systems Laboratory, National Engineering School of Monastir, Street Ibn El Jazzar, 5019 Monastir, Tunisia.*

*^*^Corresponding author email: ilyaskhan@tdt.edu.vn*

**Nomenclature**

| $C_{\infty}$ | Constant concentration | $\beta$ | | Casson parameter |
| --- | --- | --- | --- | --- |
| $T_{\infty}$ | Constant temperature | $\nu$ | | kinematic viscosity |
| $g$ | gravitational field | $\sigma$ | | Electrical conductivity |
| $Uf\left(t\right)$ | Function of velocity of plate | $B_{0}$ | | magnetic parameter |
| $\mu$ | dynamic viscosity | $\rho$ | | density of fluid |
| $\left(a,b\right)^{th}$ | component of the deformation rate |  | | parameter of porosity |
| $\pi_{c}$ | critical value of the product based on the non-Newtonian model | $k$ | | thermal conductivity |
| $\pi$ | the product of the component of deformation rate with itself | $k_{1}$ | | thermal expansion coefficient |
| $\mu_{\eta}$ | plastic dynamic viscosity of the non- Newtonian fluid | $\beta_{\Theta}$ | | Concentration expansion  coefficient |
| $P_{\lambda}$ | yield stress of fluid | $\beta_{c}$ | | Temperature expansion  coefficient |
| $C\left(y,t\right)$ | Concentration profile | $c_{p}$ | | specific heat at constant  temperature |
| $u\left(y,t\right)$ | velocity profile | $Q$ | | heat generating term |
| $\Theta\left(y,t\right)$ | temperature profile | $D$ | | mass diffusivity |
| $M$ | Magnetic parameter | $R$ | | chemical reaction parameter |
| $\Pr$ | Prandtl number | $h_{s}$ | | coefficient of convective heat transfer |
| $Sc$ | Schmidt number | $\delta$ | | heat generation parameter |
| $\Re$ | parameter of chemical reaction | $N$ | | the ratio of buoyancy force |
| $t$ | time | $\varepsilon=1$ | | MFFRP |
| $\varepsilon=0$ | MFFRF | $\lambda$ | | Newtonian heating parameter |
| $\upsilon_{m}\left(\zeta,\tau\right),$ | Mechanical components of velocity field | $\upsilon_{C}\left(\zeta,\tau\right)$ | | concentration components of the velocity field |
| $\upsilon_{\Theta}\left(\zeta,\tau\right)$ | thermal components of the velocity field | $\frac{1}{K}$ | | permeability of pours medium |
| **B** | Magnetic field |  |  | |
